# Supplementary material for: Plants from the abandoned Nacozari mine tailings: evaluation of their phytostabilization potential
Source: PeerJ. 2017 May 4;5:e3280. doi: 10.7717/peerj.3280 (PMC5420198; doi:10.7717/peerj.3280)
Supplement: Appendix S1 [file peerj-05-3280-s002.docx]

Appendix 1. - List of species recorded at the central tailing of Nacozari, Sonora. (√: Present; x: absent, N: Native; E: Non-native; I: Invasive non-native).

| Family | Scientific name |  | Patch 1 | Patch 2 | Patch 3 | Patch 4 |
| --- | --- | --- | --- | --- | --- | --- |
| Amaranthaceae | *Amaranthus watsonii* | N | √ | √ | √ | √ |
| Amaranthaceae | *Chenopodium neomexicanum* | N | √ | x | √ | x |
| Amaranthaceae | *Ambrosia confertiflora* | N | √ | √ | √ | x |
| Asteraceae | *Ambrosia ambrosioides* | N | x | √ | x | x |
| Asteraceae | *Baccharis sarathroides* | N | √ | √ | x | √ |
| Asteraceae | *Brickellia coulteri* | N | √ | √ | √ | x |
| Asteraceae | *Gnaphalium leucocephalum* | N | √ | √ | √ | x |
| Asteraceae | *Machaeranthera tagetina* | N | √ | x | x | x |
| Asteraceae | *Machaeranthera gracilis* | N | √ | x | x | √ |
| Cannabaceae | *Celtis pallida* | N | √ | √ | x | x |
| Commelinaceae | *Commelina erecta* | N | √ | x | x | √ |
| Convolvulaceae | *Ipomoea arborescens* | N | x | x | √ | x |
| Convolvulaceae | *Ipomoea cristulata* | N | x | x | √ | x |
| Convolvulaceae | *Ipomoea purpurea* | N | x | x | √ | x |
| Cucurbitaceae | *Cucurbita digitata* | N | √ | x | √ | x |
| Cyperaceae | *Cyperus elegans* | N | x | x | x | √ |
| Euphorbiaceae | *Euphorbia heterophylla* | N | √ | x | x | x |
| Euphorbiaceae | *Chamaesyce albomarginata* | N | √ | x | x | x |
| Euphorbiaceae | *Ricinus communis* | I | x | x | √ | x |
| Fabaceae | *Acacia farnesiana* | N | √ | √ | √ | √ |
| Fabaceae | *Prosopis velutina* | N | x | √ | √ | x |
| Malvacea | *Sida rhombifolia* | N | √ | x | x | x |
| Molluginaceae | *Mollugo verticillata* | N | x | x | √ | √ |
| Nyctaginaceae | *Boerhavia coulteri* | N | √ | √ | √ | √ |
| Poaceae | *Bouteloua repens* | N | √ | x | √ | x |
| Poaceae | *Bromus catharticus* | E | √ | x | x | x |
| Poaceae | *Cenchrus ciliaris* | I | x | √ | x | x |
| Poaceae | *Chloris virgata* | N | x | √ | √ | X |
| Poaceae | *Cynodon dactylon* | I | √ | √ | √ | x |
| Poaceae | *Echinochloa colonum* | E | x | x | x | √ |
| Poaceae | *Eragrostis lugens* | N | x | x | √ | x |
| Poaceae | *Panicum hirticaule* | N | √ | x | √ | x |
| Poaceae | *Setaria macrostachya* | N | √ | √ | √ | X |
| Poaceae | *Sorghum halepense* | I | x | √ | √ | √ |
| Poaceae | *Urochloa arizonica* | N | √ | x | x | x |
| Portulacaeae | *Portulaca suffrutescens* | N | √ | x | x | √ |
| Portulacaeae | *Talinum paniculatum* | N | x | √ | x | x |
| Rhamnaceae | *Ceanothus greggii* | N | x | √ | x | x |
| Solanaceae | *Datura wrightii* | N | √ | √ | x | x |
| Solanaceae | *Nicotiana glauca* | E | x | x | √ | x |
| Solanaceae | *Solanum elaeagnifolium* | N | √ | x | x | √ |
| Solanaceae | *Solanum lumholtzianum* | N | √ | x | x | √ |
