# Supplementary material for: Plants from the abandoned Nacozari mine tailings: evaluation of their phytostabilization potential
Source: PeerJ. 2017 May 4;5:e3280. doi: 10.7717/peerj.3280 (PMC5420198; doi:10.7717/peerj.3280)
Supplement: Appendix S2 [file peerj-05-3280-s003.docx]

Appendix 2. – Metal concentration (mg/kg) in leaves of the most abundant perennial species from the central Nacozari tailings deposit. Means ± standard deviation are given.

| **Element** | **Plant species** | | | | | **Maximum Tolerable**  **Level for Animals^2^**  (NRC, 2005) |
| --- | --- | --- | --- | --- | --- | --- |
|  | *Baccharis sarothroides* | *Gnaphalium leucocephalum* | *Brickellia coulteri* | *Acacia farnesiana* | *Prosopis velutina* |  |
| Ca | 17260± 7653 | 17029± 2957 | 21552± 2577 | 25795± 12737 | 49225 ± 13333 | 0.9 – 2% dry mass |
| Cu | 71.43± 15.0 | 279.49± 91.96 | 60.04± 13.64 | 124.33± 28.94 | 208.50± 48.73 | 15 – 500 mg/kg |
| Fe | 178.47±69.04 | 4456 ± 1440 | 429.76± 270.88 | 1148± 1182 | 1708±442 | 500 – 3000 mg/kg |
| K | 44976± 14042 | 73824± 6073 | 53581± 7377 | 24631± 4563 | 21899± 10608 | 1-2% dry mass |
| Mn | <BDL^1^ | 857.9± 550.4 | 320.17± 180.03 | <BDL | <BDL | 400 – 2000 mg/kg |
| Mo | 11.11± 0.83 | 17.41± 1.15 | 10.76± 0.95 | 13.88± 1.47 | 14.29±1.32 | 5 – 150 mg/kg |
| Rb | 59.23± 22.23 | 80.28± 35.13 | 51.51± 19.13 | 53.77± 7.16 | 47.30±12.59 | 200 mg/kg |
| Sr | 52.47± 35.72 | 54.13± 13.96 | 50.50± 18.34 | 80.32± 68.97 | 194.9±135.7 | 1000-2000 mg/kg |
| Zn | 277.2 ± 209.1 | 807.1 ±460.1 | 258.49± 157.72 | 159.7 ± 117.79 | 342.74±164.47 | 250-1000 mg/kg |
| Zr | 10.46± 3.61 | 16.08± 3.89 | 8.17± 0.67 | 10.17± 0.85 | 9.16±1.20 | n.a.^3^ |

^1^BDL= below detection limit

^2^Ranges represent values from the NRC (2005) report “Mineral Tolerance of Animals”. The maximum tolerable level is defined in the report as “the dietary level that, when fed for a defined period of time, will not impair animal health and performance”. Ranges are provided since values differ for animals tested including swine, poultry, horses, cattle, sheep and fish.

^3^n.a. = not available
